# A quantitative decision theory of animal conflict

**DOI:** 10.1016/j.heliyon.2021.e07621

**Published:** 2021-07-19

**Authors:** Shuang Wu, Libo Jiang, Xiaoqing He, Yi Jin, Christopher H. Griffin, Rongling Wu

**Affiliations:** a Beijing Advanced Innovation Center for Tree Breeding by Molecular Design, Center for Computational Biology, College of Biological Sciences and Technology, Beijing 100083, China; b Applied Research Laboratory, The Pennsylvania State University, University Park, PA 16802, USA; c Center for Statistical Genetics, Departments of Public Health Sciences and Statistics, The Pennsylvania State University, Hershey, PA 17033, USA

**Keywords:** Cooperation, Competition, Golden section theory, Interaction, Fish

## Abstract

Interactions between individuals are thought to shape evolution and speciation through natural selection, but little is known about how an individual (or player) strategically interacts with others to maximize its payoff. We develop a simple decision-theoretic model that generates four hypotheses about the choice of an optimal behavioral strategy by a player in response to the strategies of other players. The golden threshold hypothesis suggests that 62% is the critical threshold determining the transition of a larger player's strategy in reaction to its smaller dove-like partner. Below this critical point, the larger one exploits the smaller one, whereas above it, the larger one chooses to cooperate with the smaller one. The competition-to-cooperation shift hypothesis states that a larger player never cooperates with a smaller hawk-like player unless the former is reversely surpassed in size by the latter by 75%. The Fibonacci retracement mark hypothesis proposes that, faced with a larger dove-like player, a smaller player chooses to either cooperate or cheat, depending on whether its size relative to the larger player is less or more than 38%. The surrender-resistance hypothesis suggests that, in reaction to a larger hawk-like player, a smaller player can either gain some benefit from resistance or is sacrificed by choosing to surrender. We test these hypotheses by re-analyzing body mass data of full-sib fishes that were co-cultured in a common water pool. Pairwise analysis of these co-existing fishes broadly suggests the prediction of our hypotheses. Taken together, our model unveils detectable yet previously unknown quantitative mechanisms that mediate the strategic choice of animal behavior in populations or communities. Given the ubiquitous nature of biological interactions occurring at different levels of organizations and the paucity of quantitative approaches to understand them, results by our decision-theoretic model represent an initial step towards the deeper understanding of how biological entities interact with each other to drive their evolution.

## 1 Introduction

Organisms in communities from bacteria colonies to human societies should mutually interact in various ways to form complex behavioral relationships as they strive to acquire resources for survival and reproduction (Stachowicz 2001; Perc et al., 2013; Barraclough 2015; Jolles et al., 2020). These interactions range from antagonistic to mutualistic, including exploitation and altruism. Each of these interactions can be costly since either confrontation or cooperation requires energy (Parker and Smith 1990; Bronstein 2001; Holland 2002; Perc et al., 2017). As a general rule, to obtain a maximum payoff and fitness, each and every organism strives to choose an optimal interaction strategy such that its occupation of resources is maximized but the energy it spends is minimized (Parker and Smith 1990; Martins et al., 2017). However, the existing theory is unable to explain and, even less so, quantify when and how an organism makes such a choice.

Considerable evidence shows that phenotypic variation drives the behavior and functioning of animal interactions (Jolles et al., 2020). Animal coordinate their behavior to gain potential benefits through sensing how they phenotypically differ from others (Ward and Webster 2016; Jolles et al., 2020). Phenotypic heterogeneity that mediates collective behaviors and group-level pattern and assortment is determined by many morphological, physiological (i.e., biomechanics, energetics, and neuroendocrinology), and cognitive traits. Among all these traits that are strongly hierarchical, body size is considered as a key driving force of animal interaction strategies (Peters 1983; Calder 1984; Jolles et al., 2020). Here, we use animal size to study the choice of an optimal strategy for certain interactions. We develop a simple decision-theoretic model and apply it to a real-world problem, from which a rule of thumb is extracted to guide animal interactions.

## 2 A decision-theoretic model

**Figure 1:**
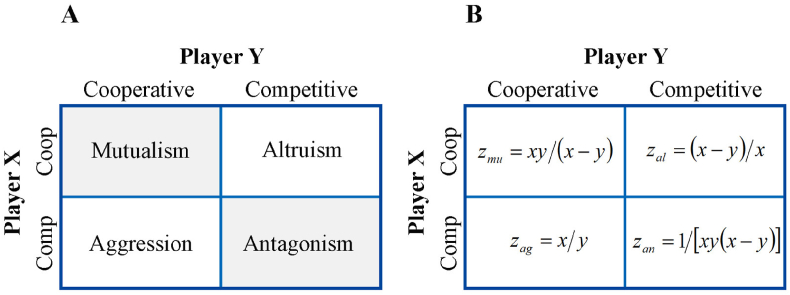
The reward matrix for two interacting players and its mathematical formulation according to behavioral ecology. (A) The matrix of strategies, triggered by two players X and Y, leading to various ecological interactions. (B) The mathematical payoff matrix of two players X and Y in terms of body size *x* and *y* (*x* > *y*) for each type of ecological interactions.

**Interaction descriptors:** Specifically, we consider an animal population in which each individual may choose to cooperate or compete with any other members, driven by resource availability or quorum sensing. Thus, any two individuals (called players) X and Y in a population will produce four types of interactions, i.e., mutualism, altruism, aggression, and antagonism (Figure 1A). We describe these interaction types based on animal size, given that animals possess basic quantitative reasoning skills (Agrillo et al., 2008).

Behavioral theory and experiments suggest that animals (such as fish or birds) of roughly similar size tend to act together as a single organism in which cooperation dominates and schooling behaviors produce an egalitarian form of organization (Axelrod 1984; Camazine et al., 2001; Nowak 2006; Sumpter 2006, 2010; Herbert-Read et al., 2011; Hemelrijk and Hildenbrandt 2012; Jiang et al., 2017). This collective motion phenomenon can avoid the oddity effect whereby individuals that differ in size and appearance from the group may incur increased risk of predation (Hoare et al., 2000; Gómez-Laplaza 2009). All these well-established theories allow us to speculate that interacting individuals’ cooperation is determined by how much they resemble each other. Since the similarity of two variables is positively correlated with their product given their fixed sum, we use the product of size traits between two individuals as a measure for the strength of mutualism. By contrast, the inverse of the product of size traits of two individuals can be approximately used to measure the strength of antagonism. To remove the scale effect, the measures of mutualism and antagonism strengths are adjusted by dividing them by the absolute difference of traits values of two animals.

Faced with the needs of foraging, reproduction, and defense from predators, dominant animals (usually in terms of body size) display aggressive and courtship behaviors upon subordinates (Chance and Larson 1976; Desjardins et al., 2012; Romenskyy et al., 2017). This agonistic behavior is adaptive, because it can enhance resource acquisition, reproduction and survival under competition for limited resources (Pan et al., 2010; Peiman and Robinson 2010; Martin et al., 2017) when a limited amount of resource needs to be allocated among different members. Based on these observations, we use the ratio of the trait values of a larger over a smaller individual as the measure of the strength of aggression. Accordingly, we use the difference between the trait values of a larger and smaller individual, divided by the larger one's value, to measure the strength of altruism.

Each type of animal-animal interaction described above can be quantified mathematically. Let x and y denote the trait values of a larger (X) and smaller animal (Y) in a pair, respectively. Based on the above definitions of interactions, we us x and y values to construct a mathematical reward matrix, describing different types of interactions between two animals, i.e., $z_{\text{mu}}$ for mutualism, $z_{\text{an}}$ for antagonism, $z_{\text{ag}}$ for aggression, and $z_{\text{al}}$ for altruism (Figure 1B).

**Figure 2:**
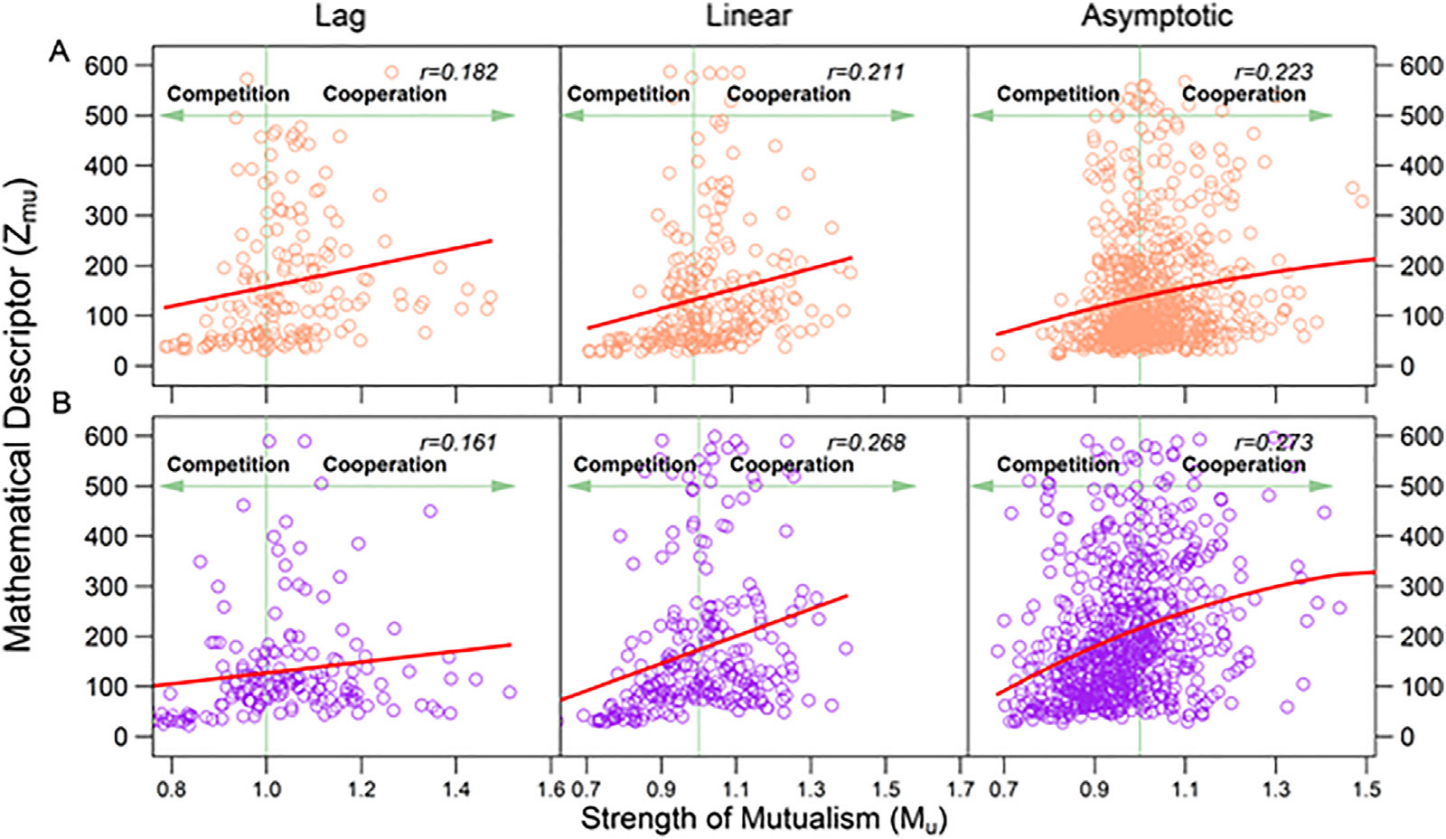
Scatter plots of mutualism descriptor (*z*_mu_) against the actual strength of mutualism (*M*_u_) across 100 interspecific pairs of strains from *E. coli* and *P. aeruginosa* (A) and *S. aureus* and *P. aeruginosa* (B) at three distinct phases of microbial growth (lag, linear, and asymptotic). The strength of mutualism is measured by the average of the ratio of abundance of each bacterial species in co-culture to monoculture. Thus, this ratio average classifies scatters into the competition region (*M*_u_ < 1) and cooperation region (*M*_u_ > 1). Dots represent observations of different interspecific strain pairs at each time point. The relationship between two variables is roughly fitted by a curve, with correlation coefficient (*r*) given within each plot.

**Biological justification:** In our previous study (Jiang et al., 2019), we used two cultural experiments for fish and bacteria to empirically justify the biological relevance of the mathematical descriptors of interaction (Figure 1B). To confirm the biological validation of these descriptors, we conducted three larger-scale cultural experiments, in each of which independent interspecific pairs of bacterial strains were reared in monoculture and co-culture. We sampled and cultured 100 pairs of *Escherichia coli* and *Pseudomonas aeruginosa* for experiment EP and 100 pairs of *Staphylococcus aureus* and *Pseudomonas aeruginosa* for experiment SP. In correspondence to x(t) and y(t) representing the abundance of a larger strain X and a smaller strain Y at time t in socialized co-culture, we use $x_{0}\left(t\right)$ and $y_{0}\left(t\right)$ to denote the abundance of X and Y at time t in socially isolated monoculture, respectively, regardless of which strain, X or Y, is more abundant in the monoculture. If two strains cooperate with each other at a time, the abundance of each strain should be greater in co-culture than in monoculture (Ojalehto et al., 2015; Ghoul and Mitri 2016). Thus, we can use $M_{u}\left(t\right)=\left(x\left(t\right)/x_{0}\left(t\right)+y\left(t\right)/y_{0}\left(t\right)\right)/2$ to quantify the strength of mutualism between strains at time *t*. By fitting and comparing three widely used growth equations, Gompertz, logistic, and Richards, to the same time-dependent abundance data, respectively, we chose an optimal one for each strain in a specific pair and then partitioned its growth curve into lag, linear, and asymptotic phases (Zwietering et al., 1990; Jiang et al., 2018). We calculated the mathematical descriptor of mutualism strength ($z_{\text{mu}}\left(t\right)$; Figure 1B) and plotted it against actual mutualism strength $\left(M_{\text{u}}\left(t\right)\right)$ across all pairs at each time point (Figure 2). On the x-axis, $M_{\text{u}}\left(t\right)$ quantifies the strength of cooperation if it is greater than 1.0 and the strength of competition if it is less than 1.0, respectively. We found that a significant positive correlation exists between $\;z_{\text{mu}}\left(t\right)$ and $M_{\text{u}}\left(t\right)$ (*P* < 0.001) over cooperation and competition regions in all three experiments EP (Figure 2A) and SP (Figure 2B). This suggests that a larger $\;z_{\text{mu}}\left(t\right)$ value is associated with a smaller degree of competition or with a larger degree of cooperation, respectively, in the situation where two microbes compete or cooperate. Taken together, we can use $\;z_{\text{mu}}\left(t\right)$ as an effective proxy of mutualism measure, especially when the growth of strains reaches its linear and asymptotic phases.

**Figure 3:**
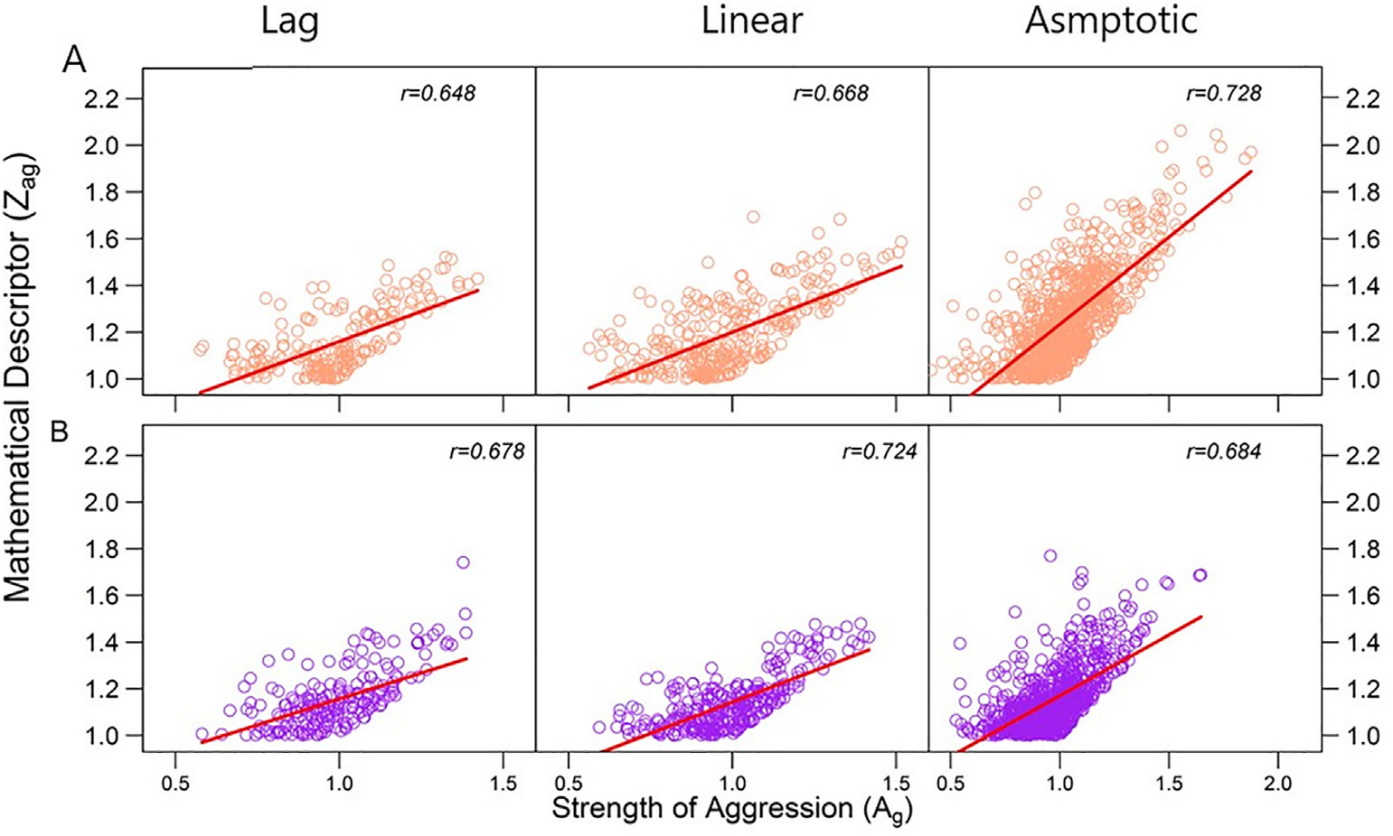
Scatter plots of aggression descriptor (*z*_al_) against the actual strength of aggression (*A*_g_) across 100 interspecific pairs of strains from *E. coli* and *P. aeruginosa* (A) and *S. aureus* and *P. aeruginosa* (B) at three distinct phases of microbial growth (lag, linear, and asymptotic). Dots represent observations of different interspecific strain pairs at each time point. The relationship between two variables is roughly fitted by a curve, with correlation coefficient (*r*) given within each plot.

**Figure 4:**
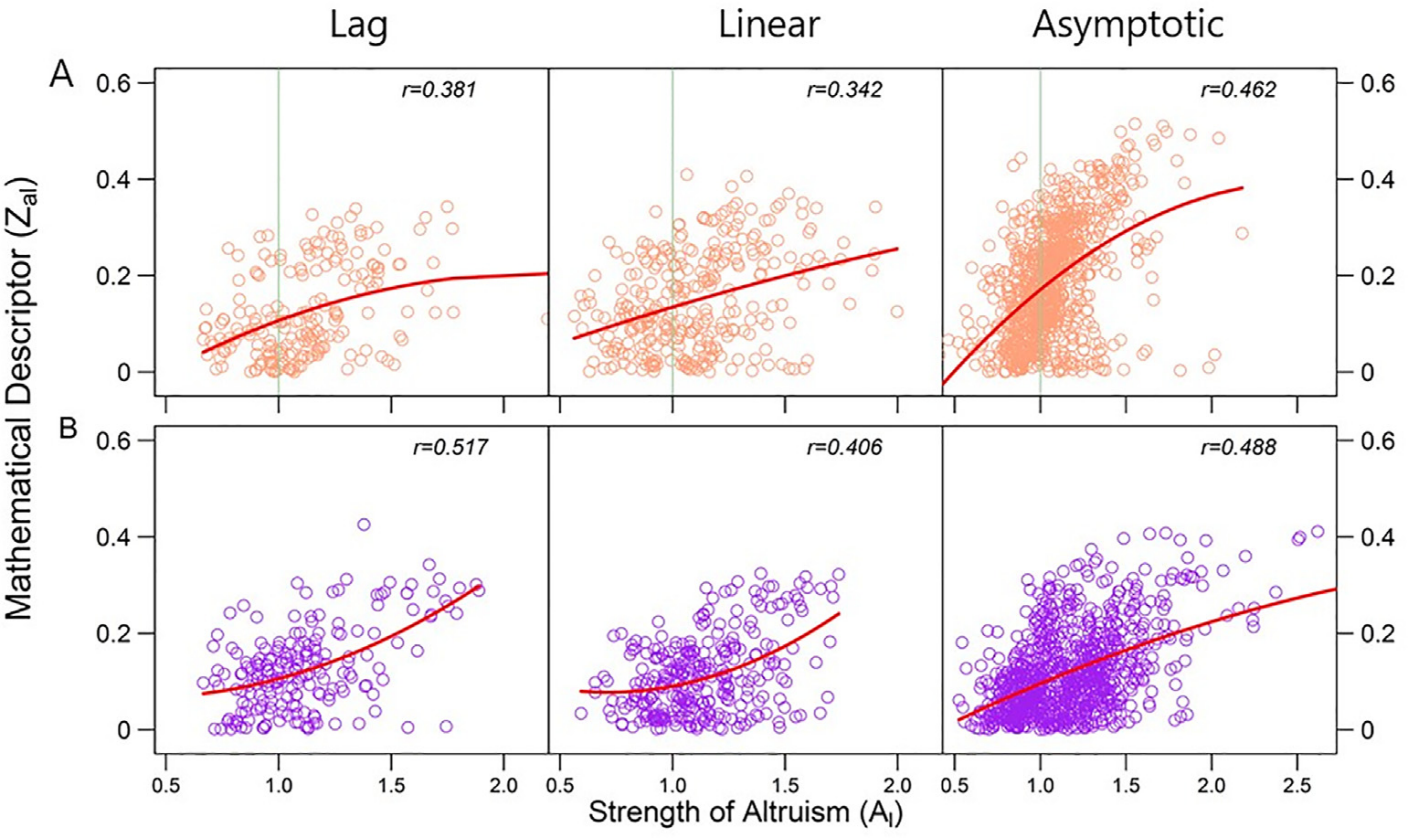
Scatter plots of altruism descriptor (*z*_ag_) against the actual strength of altruism (*A*_l_) across 100 interspecific pairs of strains from *E. coli* and *P. aeruginosa* (A) and *S. aureus* and *P. aeruginosa* (B) at three distinct phases of microbial growth (lag, linear, and asymptotic). Dots represent observations of different interspecific strain pairs at each time point. The relationship between two variables is roughly fitted by a curve, with correlation coefficient (*r*) given within each plot.

If one strain is aggressive on the other, i.e., the former grows at a cost of the latter, then the relative abundance of the former over the latter should increase through some regulatory systems when the two strains are relocated from their respective isolated environments to the socialized environment (Gonzalez and Mavridou 2019). By contrast, if one strain is altruistic upon the other, i.e., the former sacrifices itself to benefit the latter (Lewin-Epstein et al. 2017), then the relative abundance of the latter in co-culture over monoculture should be larger than the relative abundance of the former in co-culture over monoculture (Kreft 2004). Based on these arguments, we can define $A_{g}\left(t\right)=\left(x\left(t\right)/y\left(t\right)\right)/\left(x_{0}\left(t\right)/y_{0}\left(t\right)\right)$ and $A_{l}\left(t\right)=\left(y\left(t\right)/y_{0}\left(t\right)\right)/\left(x\left(t\right)/x_{0}\left(t\right)\right)$ to quantify the strength of a larger strain's aggression upon a smaller strain and the strength of altruism at time t, respectively. We found that all three experiments support significant positive correlations of $z_{\text{ag}}\left(t\right)$ with $A_{g}\left(t\right)$ (Figure 3; *P* < 0.001) and of $z_{\text{al}}\left(t\right)$ with $A_{l}\left(t\right)$ (Figure 4; *P* < 0.001), indicating the effectiveness of $z_{\text{ag}}\left(t\right)$ and $z_{\text{al}}\left(t\right)$ as a proxy to measure the strengths of aggression and altruism, respectively.

Our previous cultural experiments using different species have well supported the biological relevance of Figure 1B's mathematical descriptors as measures of the strengths of different types of interactions (Jiang et al., 2019). In our current experiments by culturing multiple species combinations with much larger sample sizes, such relevance has been reaffirmed. These biologically meaningful descriptors allow us to frame novel hypotheses regarding the strategical choice of animals to pursue reciprocal interactions.

**How a larger player reacts to a smaller player?** Under the assumption x>y, analysis of animal behavior when they are very close in size results in undefined division by zero. Therefore, all rewards (Figure 1B) can be relativized with respect to the aggression/aggression behavior to obtain the well-behaved reward matrix:

(1) $$\left(\begin{array}{cc} x^{2}y^{2} & {\left(x-y\right)}^{2}y\\ x^{2}\left(x-y\right) & 1 \end{array}\right)$$

In general, we assume X makes decisions in rows, while Y makes decisions in columns. For varying values of x and y, this matrix will either reward aggression or non-aggression in the pair of players. It is worth noting that this matrix changes depending on which player is interacting.

We consider lower-order animals without a fully developed theory of cognition. We hypothesize that such players are not making decisions based on absolute size, but relative size. We can therefore analyze the break-even point if a larger player is confronted with a smaller player that act in a non-aggressive manner (dove). Assuming some decision making ability, the larger player is non-aggressive if

$$x^{2}y^{2}>x^{2}\left(x-y\right)$$

Identifying the break-even leads to the simple quadratic equation:

$$y^{2}+y-x=0,$$

or

(2) $$y=\frac{1}{2}\left(-1+\sqrt{1+4x}\right).$$

Considering the smaller player's behavior and relativizing to the larger player (i.e., setting x=1), Eq. (2) implies that

(3) $$y=\frac{1}{2}\left(-1+\sqrt{1+4}\right)=\phi ,$$

where *ϕ* is the golden ratio. This is the first *testable hypothesis*: when a larger player encounters a smaller dove-like player, it acts aggressively when the smaller player is below 62% of its size and cooperatively when it is larger than that. In other words, the golden ratio is a cut-off point that helps a player chooses cooperation or conflict. If a larger player still chooses to compete with a smaller dove-like player when the latter's size is beyond 62% of the former's size, both, especially the larger one, need to invest extra energy.

Based on the reward matrix (1), the break-even point at which a larger player reacts to a smaller aggressive player (hawk) can be obtained by solving the equation

$${\left(x-y\right)}^{2}y=1$$

whose solution is *y* ≈ 1.75 by letting *x* = 1. Given 0<y<x=1, this produces a second *testable hypothesis*: a larger player should *never* act non-aggressively to a smaller hawk-like player, i.e., there is never an incentive to not react to aggression with aggression. More precisely, a larger player may not cooperate with a smaller player that acts aggressively unless the latter grows to surpass in reverse the former by 75%. Before reaching this percentage, the larger player will always be aggressive upon the smaller hawk.

**How a smaller player reacts to a larger player?** Based on the reward matrix (1), this question can be answered by detecting the “break-even” points of the following equations:

(4A) $$x^{2}y^{2}={\left(x-y\right)}^{2}y$$

(4B) $$x^{2}\left(x-y\right)=1$$

which is simplified as *y*^2^ – *x*^2^*y* – 2*xy* + *x*^2^ = 0. Let *x* = 1 by normalization and, then, we have *y*^2^ – 3*y* + 1 = 0, whose non-extraneous solution is

(5) $$y=\frac{1}{2}\left(3-\sqrt{5}\right)=1-\phi =0.382.\;$$

If the relative trait value of player Y to X is smaller than the 38%, the *Fibonacci retracement mark*, the smaller player Y prefers to cooperate with the larger dove-like player X. Yet, if this relative value is larger than 38%, player Y becomes interested in acquiring benefit from player X by cheating the latter, which is a third *testable hypothesis.* From Eq. (4B), we solve *y* = 0 by letting *x* = 1, which generates a fourth *testable hypothesis*. Faced with the larger hawk-like player, the small player would be sacrificed if it chooses to cooperate, but it may benefit from its choice of conflict with the larger player.

## 3 Experimental validation

**Figure 5:**
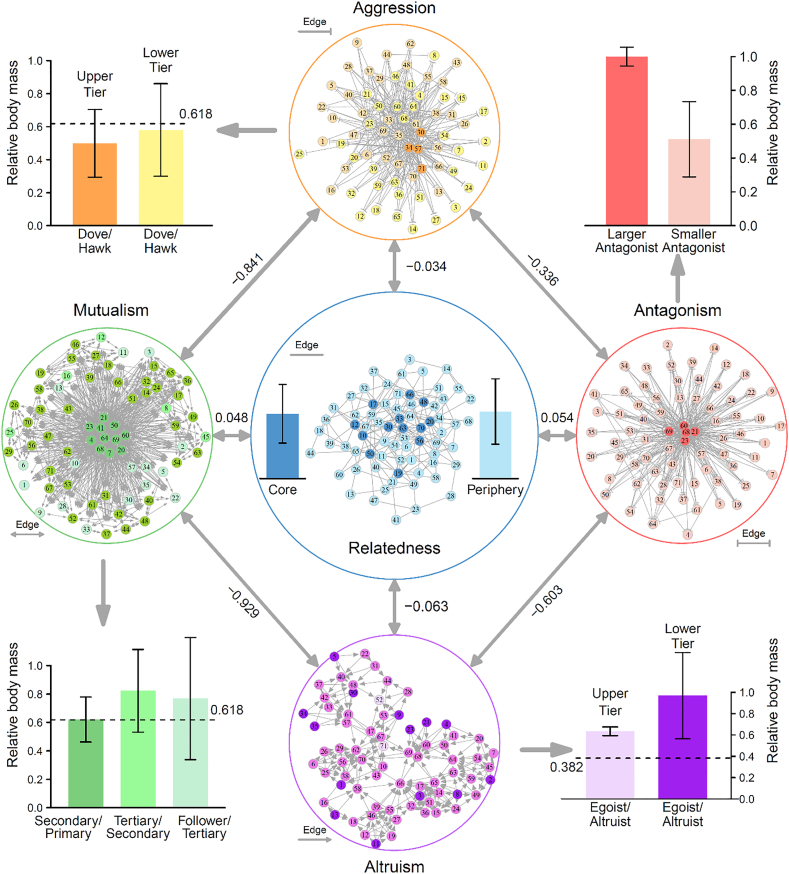
Social networks of all fish growing in a society from a full-sib family. Left: Mutualism network constructed by doubly-arrowed edges representing fish-fish cooperation. Right: Antagonism network composed of doubly-T-shaped edges denoting the mutual conflict of the fish. Top: Aggression network represented by singly-T-shaped edges specifying how a fish (as a hawk) aggresses upon others (doves) (color metrics indicate three hierarchies of aggression). Bottom: Altruism network characterized by singly-arrowed edges which illustrates how a fish (altruist) benefits other fishes (egoists) at its own expense (color metrics indicate three hierarchies of altruism). Center: The relatedness network constructed from genome-wide marker data, which contains core members (because of their similarity to a large number of siblings) and peripheries. In each network, we indicate different members based on the metric of color and compare their relative body mass. Network similarity is described by the coefficients of correlation calculated over different fish pairs. The identity of each fish is labeled by a number.

We reanalyze a published data from a mapping experiment of fish (*Cyprinus carpio*) conducted with a full-sib family composed of 71 full-sibs co-cultured in a common water pool. The fish have schooling and aggregation behaviors, making them an ideal model system to investigate how social interactions impact phenotypic differentiation (Jiang et al., 2017). Using interaction descriptors, we constructed four 71-node networks of interactions, i.e., mutualism, altruism, aggression and antagonism (Figure 5). In the mutualism network, we identified hub fish, i.e., those whose links are much more than average, and named them as primary leaders. The fish that are directly linked by the primary leaders are called secondary leaders. Those whose routes to the primary leaders are separated by the secondary leaders are regarded as tertiary leaders. The fish that are linked by the tertiary leaders but not linked by the secondary leaders are called the followers. We calculated the relative body mass of the secondary leaders over the primary leaders, which is averaged 0.621 over all pairs (left at the bottom, Figure 5). Similarly, the relative body mass of the tertiary leaders over the secondary leaders and the secondary leaders over the followers were calculated, averaged 0.823 and 0.767, respectively. All these relative values are larger than 0.618, especially at a significant level for the latter two (*P* < 0.001), which is in agreement with the first hypotheses that a larger fish tends to establish a mutualistic relationship with a smaller cooperative member if the small-large ratio is beyond the golden ratio.

The aggression network is composed of two hierarchical organizations: the aggressive group (hawks) and the submissive group (doves). Some fish may play both hawks and doves if they are aggressive to one member but submissive to another member. By comparing the body mass of hawks and doves in the network, we found that the ratio of the hawks-doves to the hawks is 0.502 (the upper tier) whereas the ratio of the doves to the hawks-doves is 0.581 (the lower tier), with both ratios being significantly lower than 0.618 (*P* < 0.05) (left at the top, Figure 5). This amount of difference complies with the first hypothesis that a larger individual would exploit a smaller dove-like member when the relative size of the latter over the former is less than the golden ratio.

The altruism network includes altruists that provide benefit to others and egoists that receive benefit from altruists (right at the bottom, Figure 5). Some fish serve as both altruist and egoists. The relative body mass of egoists over altruists is 0.634 at the upper tier (egoists-altruists vs. altruists) and 0.962 at the lower tier (egoists vs. egoists-altruists vs. altruists), both being significantly larger than 0.382 (*P* < 0.05). This is consistent with the third hypothesis that egoists start to cheat altruists when their relative strength over the latter is beyond the Fibonacci retracement mark. In the antagonism network, each pair of nodes is linked by two antagonists (right at the top, Figure 5). In general, hubs in this network are a group of smaller fish. We calculated the relative body mass of smaller antagonists over larger antagonists, which is 0.510. This number appears to be consistent with the fourth hypothesis that the smaller antagonists can obtain some benefit from the larger antagonists when the former adopt a tit-for-tat strategy to the latter.

Despite being from a full-sib family, a pair of fish as siblings may still differ in genetic relatedness since they inherit the same alleles from the parents at different likelihoods. We calculated the coefficients of genetic similarity based on genome-wide segregating SNPs and used them to construct a relatedness network (center, Figure 5). We did not detect any significant difference in body mass between hub fish (core members) and other fish (peripheral members) (*P* = 0.45). Correlation analysis of networks suggests that the pattern of social interactions does not depend on how much genetic similarity there is between the fish. Both mutualism and antagonism as bidirectional interactions are negatively associated with directional altruism and aggression, respectively (*P* < 0.001). Taken together, whether a fish chooses a competitive or cooperative strategy in response to its conspecifics is not associated with their genetic relatedness, rather than with their relative body size.

## 4 Discussion

Our decision-theoretic model produces four testable hypotheses regarding how one animal interacts with others to maximize its payoff. The first hypothesis, called the golden section threshold hypothesis, states that a larger animal is aggressive upon a smaller nonaggressive animal if their relative smaller-to-larger size is less than 62%, and otherwise, it would decide to cooperate when the relative size is beyond this number. This hypothesis can be explained by the optimal ratio of benefit and cost (Parker and Smith 1990; Bronstein 2001; Holland 2002). For example, a bigger animal only needs less cost to exploit a smaller animal, but this exploitation will become extremely costly if the smaller animal reaches or outmatches the golden ratio relative to the larger animal. The golden ratio of two quantities, intriguing mathematicians for at least 2,400 years, has been observed to occur in ancient and modern architecture, sculpture and painting (Livio 2002), and has been recently claimed to approach the relative masses of two quasiparticles in cobalt niobate under a particular magnetic field (Affleck 2010). It has been believed to also occur pervasively during biological morphogenesis, although its biological relevance is far from being well explained (Henein et al., 2011; Persaud and O'Leary 2015).

Encountered with a smaller but aggressive animal, a larger animal will never be non-aggressive, but if the smaller animal grows to reversely surpass the larger animal by 75%, the larger one will have no choice but cooperate. This is a second hypothesis, called the competition-to-cooperation shift hypothesis. We name the third hypothesis the Fibonacci retracement mark hypothesis. According to this hypothesis, while a smaller animal benefits from a larger nonaggressive animal through cooperation, it may become cheating to be aggressive upon its benefactor when its relative size outmatches the Fibonacci retracement mark. It is worth noting that Fibonacci retracement also emerges naturally in financial analysis as heuristic strategy for stock management (Lim 2016). The fourth hypothesis, called the resist-surrender hypothesis, is that faced with a larger aggressive animal, a smaller animal can either gain some benefit from resistance or is sacrificed by choosing to surrender.

These four testable hypotheses were proposed through a heuristic mathematical analysis of ecological phenomena to explain how different organisms choose their optimal behavioral strategies in response to the strategies of their conspecifics in a population or community. We cultured full-sib fish from a mapping population to validate these hypotheses. The first and third hypotheses can be well tested, while the fourth hypothesis partially tested. As an open question, testing the second hypothesis needs a longitudinal experiment of culture, from which dynamic interactions can be monitored.

The premise of our hypothesis formulation is the mathematical descriptors of interaction types derived from animal behavioral ecology. Collective behavior and social grouping are omnipresent phenomena in the animal kingdom, ranging from pairs of individuals to communities (Axelrod 1984; Sumpter 2010; Herbert-Read et al., 2011; Gómez-Laplaza 2009). The mechanisms underlying these phenomena are the strategies of how animals sense, perceive, and cope with others to gain their maximum benefits, such as increased mating opportunities, improved foraging efficiency, lower predation risk, and reduced energetic costs (Krause and Ruxton 2002; Ward and Webster 2016; Martin et al., 2017; Romenskyy et al., 2017). Body size is a fundamental trait that determines the strategies of animal interactions (Peters 1983; Calder 1984; Jolles et al., 2020). We derived a series of mathematical descriptors of animal interactions based on animal size. These descriptors have been biologically validated and justified by cultural experiments using multiple species, showing their conceptual compatibility and complementarity to fundamental ecological theory. There are also many other traits modulating interaction strategies, such as colors, scents, physiological state, learning and experience, age, cognition, and territoriality. Different mathematical descriptors are needed to characterize animal behavior based on these traits.

Our hypotheses will not only stimulate new thinking about ecological interactions and their resulting evolution and speciation, but also may hold potential implications for cancer cell biology, social network science, and even international political science. For example, a tumor may be composed of many size-varying, interacting cell populations, which can be distinguished by modern single-cell analysis techniques (Stanta and Bonin 2018). For two cooperative intratumoral cell populations, a priority should be given to control the growth of the smaller cell population to a range of 38–62% of the larger population, in which case two types of cell populations shift their cooperation to competition. This simplified intuition may provide a start point to convey important concepts and rules of thumb for complex biological or even societal systems.

## 5 Methods

### 5.1 Validation experiment of interaction descriptors

In Figure 1B, we provide four mathematical interaction descriptors that are used to measure the strengths of mutualism, antagonism, aggression, and altruism based on the body size of two pairing animals. To justify the biological relevance of these descriptors, we conducted a cultural experiment using two bacterial species. We collected 100 diverse strains from each of three bacterial species, *Escherichia coli* (E), *Staphylococcus aureus* (S), and *Pseudomonas aeruginosa* (P), and grew each strain from each species in monoculture and two interspecific pairs (EP and SP) with two randomly selected strains each from a different species in co-culture. We used quantitative PCR (qPCR) technique to measure the abundance of each strain in both monoculture and co-culture experiments once every two hours during the first 24 h, followed by once every four times till 36 h, after the cultural experiment started.

Microbial growth is thought to obey some biological rule described by growth equations (Zwietering et al., 1990). We used three commonly used Gompertz, logistic, and Richards equations to fit the time-dependent change of abundance for each strain in its socially isolated condition and socialized condition, from which an optimal one was chosen in each case through statistical reasoning, such as F-test (Jiang et al., 2019). Based on the growth equation chosen, we determined the lag, linear, and asymptotic phases of microbial growth (Zwietering et al., 1990). By comparing the abundance change of the same strain in co-culture and monoculture, we can determine how it responds to its co-existing member in a socialized environment through cooperation or competition. This comparison enables the calculations of the actual strengths of mutualism (*M*_u_), antagonism (*A*_n_), aggression (*A*_g_), and altruism (*A*_l_) for each time point at each phase in co-culture.

### 5.2 Validation experiment of decision hypotheses

We used a published genetic mapping data of fish (Jiang et al., 2019) to test our hypotheses. The mapping population is a full-sib F_1_ family of *Cyprinus carpio* including 71 progeny produced by Hebao Red carp and Koi carp. The fish were cultured in the common water pool at the Research Institute for Heilongjiang River Fisheries, Harbin, China, and measured for body mass after anesthesia with MS222 when they reached an adult stage of fish growth. As a fitness-related trait, we used body mass as a measure of the payoff of decision making by each fish. For a given pair of fish, whose body mass is *x* and *y* (*x* > *y*), we calculated the mathematical descriptors of four interaction types, mutualism, altruism, aggression and antagonism, based on the reward matrix (Figure 1B). Considering all possible pairs, we calculate *n*(*n* – 1)/2 descriptor values for each type of interaction. By culling off a proportion of small values, we reconstruct four n-node sparse networks, each for a different type of interaction. Jiang et al. (2019) reported 39,960 Mendelian segregating SNPs throughout the common carp genome of size ~1.42 Gb after quality control for this F_1_ family. These SNPs were used to calculate the degree of pairwise relatedness among 71 full-sibs.

## Declarations

### Author contribution statement

Shuang Wu & Libo Jiang: Analyzed and interpreted the data.

Xiaoqing He & Yi Jin: Performed the experiments.

Christopher H. Griffin: Analyzed and interpreted the data; Wrote the paper.

Rongling Wu: Conceived and designed the experiments; Wrote the paper.

### Funding statement

This work was supported by 10.13039/100000001National Science Foundation (DMS-1814876) (CHG).

### Data availability statement

Data associated with this study has been deposited at https://github.com/BeijingCCB/GoldenTheory for public use.

### Declaration of interests statement

The authors declare no conflict of interest.

### Additional information

No additional information is available for this paper.
